# Landscape factors influencing the distribution of rare submerged plant species: an environmental DNA (eDNA) study

**DOI:** 10.7717/peerj.21096

**Published:** 2026-04-21

**Authors:** Ayaka Fujiwara, Kei Uchida, Satoshi Yamamoto, Hirotoshi Sato, Ryohei Nakao, Masayuki K. Sakata, Norio Hayashi, Hiroki Yamanaka, Atushi Ushimaru, Toshifumi Minamoto

**Affiliations:** 1 Graduate School of Human Development and Environment, Kobe University, Kobe, Hyogo, Japan; 2 Faculty of Environmental Studies, Tokyo City University, Tokyo, Japan; 3 Institute for Agro-Environmental Sciences, National Agriculture and Food Research Organization, Tsukuba, Ibaraki, Japan; 4 Faculty of Advanced Science and Technology, Ryukoku University, Otsu, Shiga, Japan; 5 Graduate School of Human and Environmental Studies, Kyoto University, Kyoto, Kyoto, Japan; 6 Graduate School of Sciences and Technology for Innovation, Yamaguchi University, Ube, Yamaguchi, Japan; 7 Research Faculty of Agriculture, Hokkaido University, Sapporo, Hokkaido, Japan; 8 Natural History Museum and Institute, Chiba, Chiba, Japan

**Keywords:** *Najas*, Submerged plants, Environmental DNA (eDNA), Habitat, Landscape, Conservation

## Abstract

Effective conservation of rare submerged plant species requires understanding not only their habitats but also the surrounding landscape and water quality conditions. However, conventional surveys are often difficult due to the elusive nature of these plants. In this study, we applied environmental DNA (eDNA) analysis to detect species of the genus *Najas*, several of which are classified as endangered in Japan. We conducted field surveys and eDNA analysis across 158 ponds in western Japan and evaluated the influence of environmental and landscape variables using generalized linear models. Explanatory variables included water quality (pH, dissolved oxygen, electrical conductivity), pond size, shoreline type (soil, forest, concrete), and land use (forest, paddy field, artificial land). Our results showed that *Najas* presence was positively associated with lower electrical conductivity and longer forested shorelines. These findings suggest that forested pond margins may provide favorable conditions for submerged plants, while eutrophication may reduce habitat suitability. Our study demonstrates that integrating eDNA detection with landscape analysis offers a powerful and efficient approach for monitoring and conserving rare submerged plant species.

## Introduction

To conserve biodiversity and protect habitats effectively, it is important to consider not only where species live but also the environmental conditions of their habitats and surrounding landscapes. Traditionally, conservation strategies often begin by surveying the distribution of flagship species, aiming to protect entire ecosystems. However, such approaches may overlook the critical influence of habitat and landscape conditions, which are essential for the survival of target species. Once these environments are degraded, the species may no longer be able to inhabit them. Therefore, conservation planning should incorporate not only the ecological characteristics of target species but also the conditions of their habitats and surrounding environments.

Aquatic ecosystems—including seas, lakes, and rivers—are vital habitats that support diverse aquatic organisms. However, surveying these ecosystems is often more challenging, labor-intensive, and potentially hazardous than terrestrial surveys due to limited visibility and accessibility in water, sometimes requiring entering the water (Haga & Ishikawa, 2014). These difficulties, such as the lack of effective sampling tools for submerged aquatic plants, are especially challenging because these species are immobile and often occur at low densities in aquatic environments. Despite their importance for biodiversity, information on the distribution of rare submerged plants remains scarce.

Environmental DNA (eDNA) analysis has emerged as a powerful and efficient tool for surveying rare aquatic species (Ficetola et al., 2008). It is now widely used in aquatic ecology and has been successfully applied across various taxonomic groups (Minamoto, 2022; Takahashi et al., 2023; Johnson et al., 2023). Recent studies have expanded the application of eDNA to plants, including aquatic species, thereby demonstrating its potential for biodiversity monitoring and conservation. Reviews emphasize that primer selection and multi-locus strategies are critical for accurate detection, and several works have successfully applied eDNA to macrophytes in freshwater ecosystems (Johnson et al., 2023; Banerjee et al., 2022). For submerged plant surveys, eDNA offers significant advantages over conventional methods, allowing researchers to collect water samples and analyze them safely without the need for underwater fieldwork (Matsuhashi et al., 2016; Inui et al., 2018). These advances highlight the growing importance of eDNA in addressing knowledge gaps for rare submerged plants. Building on this progress, our study integrates eDNA detection with landscape analysis to identify environmental factors influencing the distribution of *Najas* species in agricultural ponds.

In this study, we focus on submerged species of the genus *Najas*, which are ecologically understudied due to their rarity and underwater habitat. These plants inhabit lentic freshwater environments such as lakes, ponds, and paddy fields (Kadono, 2014). The genus *Najas* includes nine species: *Najas marina*, *Najas tenuicaulis*, *Najas minor*, *Najas oguraensis*, *Najas gracillima*, *Najas yezoensis*, *Najas graminea*, *Najas ancistrocarpa*, and *Najas orientalis*. More than half of these species (*N. tenuicaulis*, *N. minor*, *N. gracillima*, *N. yezoensis*, *N. ancistrocarpa*, and *N. orientalis*) are listed as endangered or threatened in Japan’s national Red List (Ministry of Environment, Government of Japan, 2020; Table S1). Field surveys are often hindered by the inconspicuous nature of these plants, which lack distinctive flowers and require mature seeds for accurate identification, making taxonomic work seasonally dependent (Kadono, 1994).

To develop a conservation strategy for *Najas* species, we investigated both their habitats and surrounding environments. We hypothesized that combining eDNA detection with environmental and landscape data would help identify key factors influencing the distribution of these rare species. For example, eutrophication can lead to increased phytoplankton, which raises electrical conductivity (EC) and reduces light availability, thereby limiting suitable habitats for submerged plants (Zhang et al., 2021). Integrating eDNA analysis with aquatic and landscape surveys offers a promising approach to clarify the essential habitat requirements of this rare plant group.

Our objective was to determine how aquatic and landscape factors influence the distribution of rare submerged plant species by combining eDNA analysis with field surveys and GIS-based landscape assessments. We developed an eDNA assay for *Najas* and used it to reveal their spatial distribution across a broad geographic area. This approach represents an effective method for surveying rare aquatic plants and analyzing the relationship between their distribution and environmental variables. Due to the rarity and underwater habitat of *Najas*, distribution data are limited, impeding conservation efforts. By combining eDNA analysis with landscape and water quality assessments, our study provides new insights into the ecology and conservation of these elusive species.

## Materials & Methods

### Primer design for the genus *Najas*

To design primers for *Najas* species, we preliminarily compared several genetic regions: maturase K (matK), ribulose-1,5-bisphosphate carboxylase/oxygenase (rbcL), the trnH–psbA intergenic spacer, and internal transcribed spacers (ITS1 and ITS2) between ribosomal RNA genes. Sequences were obtained from the National Center for Biotechnology Information (NCBI). After preliminary consideration, we selected ITS1, located between the 18S and 5.8S rRNA genes, as the target region because of its interspecific variation among *Najas* species and the favorable GC content of the designed primers. Next, several primer sets were manually designed based on the target sequences, and the final set was selected using Multiple Primer Analyzer (Thermo Fisher Scientific). The selected primer sequences are listed in Table 1 (Forward primer: Tm = 59.5 °C, GC = 52.6%; Reverse primer: Tm = 59.2 °C, GC = 52.4%).

**Table 1:** Sequences of primers used in this study.

Name	Sequence (5′–>3′)	Tm (°C)	Amplicon length (bp)	Reference
Plants-NuITS1-F	ACCTGCGGAAGGATCATTG	59.5	∼220	This study
Plants-NuITS1-R	AGATATCCGTTGCCGAGAGTC	59.2		This study
1st PCR forward primer	ACACTCTTTCCCTACACGACGCTCTTCCGATCT-NNNNNNACCTGCGGAAGGATCATTG		∼300	This study
1st PCR reverse primer	GTGACTGGAGTTCAGACGTGTGCTCTTCCGATCT-NNNNNNAGATATCCGTTGCCGAGAGTC			This study
2nd PCR forward primer	AATGATACGGCGACCACCGAGATCTACAC-XXXXXXXXCACTCTTTCCCTACACGACGCTCTTCCGATCT		∼450	Miya et al. (2015)
2nd PCR reverse primer	CAAGCAGAAGACGGCATACGAGAT-XXXXXXXXGTGACTGGAGTTCAGACGTGTGCTCTTCCGATCT			Miya et al. (2015)

**Notes.**

Tm values are provided only for primers without adapters.

Eight consecutive Xs indicate index sequences.

PCR, polymerase chain reaction.

### *In silico* and *in vitro* assessment of the primers

We performed an *in silico* assessment using Primer-BLAST to verify whether the designed primers correctly amplify species belonging to the genus *Najas*. In addition, to evaluate the effectiveness of the amplified region as a DNA barcode, we calculated sequence dissimilarity for the region amplified by the primers using the *dist.dna* function in the R package *ape*.

For the *in vitro* assessment, we tested the usefulness of the primers by polymerase chain reaction (PCR) using tissue DNA from target species. Samples of *N. marina*, *N. tenuicaulis*, *N. gracillima*, and *N. graminea* were collected in the field. *N. minor*, *N. oguraensis*, and *N. yezoensis* were provided by Dr. Yasuro Kadono (Kobe University), and *N. ancistrocarpa* was obtained from a cultivated specimen at the Natural History Museum and Institute, Chiba. DNA was extracted using the DNeasy Plant Mini Kit (QIAGEN, Hilden, Germany). PCR reactions (25 µL) contained 1 × Ex Taq buffer (Takara Bio), 0.2 mM dNTPs, 0.625 U Ex Taq HS (Takara Bio Inc.), 0.4 µM of each primer, and 1 µL of template DNA. PCR conditions were: initial denaturation at 94 °C for 2 min, followed by 35 cycles of 94 °C for 30 s, 55 °C for 30 s, and 72 °C for 45 s. Amplified fragments were visualized using 1% agarose gel electrophoresis.

### Field surveys

**Figure 1:**
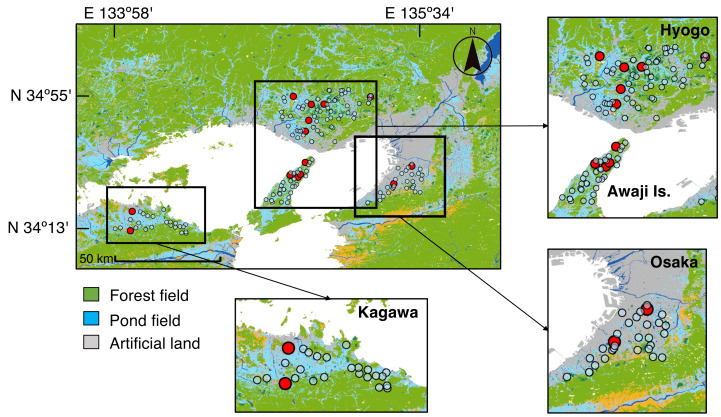
Results of eDNA detection from water samples collected at 158 ponds. Red circles indicate positive detections of *Najas* species, while transparent circles indicate negative detections. The map was created using ArcMap 10.8 (Esri Japan) based on high-resolution land-use mesh data provided by the Ministry of Land, Infrastructure, Transport and Tourism (MLIT), Japan, which is open-source and freely available (https://nlftp.mlit.go.jp/ksj/gml/datalist/KsjTmplt-L03-b.html).

Verbal permission for the field surveys was obtained on-site from the owner or rights holder of each pond. These informal approvals, consistent with local practices, were granted at the time of the visits. As the surveys involved non-invasive sampling in privately managed agricultural ponds, no formal written permissions or institutional approvals were required. We surveyed 158 ponds: 102 in Hyogo Prefecture (including Awaji Island), 25 in Kagawa Prefecture, and 31 in Osaka Prefecture (Fig. 1, Table S2). Hyogo and Kagawa have the highest and third-highest numbers of agricultural irrigation ponds in Japan, respectively. Although Osaka has fewer ponds, its pond density per unit rice paddy area ranks third, making all three prefectures representative of high-density irrigation pond regions (Uchida, 2003). Awaji Island was treated as a separate region in statistical analyses because it is geographically isolated.

Ponds were selected to represent a wide range of characteristics, including pond size, shoreline type (soil, forest, or concrete), and surrounding land use (forest, paddy field, or artificial land). Aquatic parameters—pH, dissolved oxygen (DO), and EC—were measured at one shoreline point per pond using a multiparameter water quality meter (WQC-24, DKK-TOA). Pond size and shoreline types were quantified using Japonyol (http://japonyol.net/editor/calculate.html), and land use within a 500 m radius was assessed using ArcGIS 9 (ArcMap v9.3, Esri).

We collected 1 L of water from each pond using a pre-bleached plastic bottle, added one mL of 10% benzalkonium chloride to prevent DNA degradation (Yamanaka et al., 2017). The samples were transported to the laboratory at ambient temperature and filtered twice (500 mL each) through glass-fiber filters (47 mm diameter, GF/F; GE Healthcare Life Sciences, Little Chalfont, UK), for a total of 1,000 mL per pond. Filters from each pond were combined for DNA extraction. Most samples were filtered on the same day of collection. For samples from remote locations far from the laboratory, filtration was performed 1–4 days after collection. One blank control was prepared per survey day. All equipment was sterilized with commercial bleach to prevent contamination. Filters were stored at −25 °C for up to six months prior to DNA extraction.

### DNA extraction of field samples

Throughout all laboratory procedures, the experimenter wore disposable gloves. All instruments that contact samples were either disposable or decontaminated using commercially available sodium hypochlorite solution. Extraction of eDNA and PCR setup were conducted in a dedicated laboratory for eDNA experiments, separate from rooms where tissue DNA or PCR amplicons were handled. PCR experiments were performed in a different room designated exclusively for PCR and post-PCR processes.

DNA extraction followed the protocol of Uchii, Doi & Minamoto (2016) using the DNeasy Blood & Tissue Kit (QIAGEN) and Salivette tubes (Sarstedt, Tokyo). Samples collected on the same day were processed in the same extraction batch. One negative control (extraction blank) was added to each DNA extraction batch. The following steps were performed according to Uchii, Doi & Minamoto (2016): two filters were placed in a Salivette tube with 400 µL Buffer AL and 40 µL Proteinase K, incubated at 56 °C for 30 min, and centrifuged at 3,000× g for 3 min. Then, 220 µL TE buffer was added, allowed to stand for 1 min, and centrifuged again for 1 min. The eluate was mixed with 400 µL absolute ethanol and transferred to a DNeasy Mini Spin Column, centrifuged at 6,000× g for 1 min, and repeated until all solution was processed. Subsequent steps followed the manufacturer’s manual, and DNA was eluted in 100 µL and stored at −25 °C until PCR.

### Library construction

First-round PCR (10 µL) included 1 × PCR buffer, 0.2 mM dNTPs, 1.5 mM MgSO_4_, 0.3 µM of each primer (Table 1), 0.2 µL KOD-Plus-Neo (TOYOBO), and 1–2 µL template DNA. Three negative controls were included per PCR run. Conditions: 94 °C for 2 min, 98 °C for 10 s, 35 cycles of 59 °C for 30 s and 68 °C for 30 s, final extension at 68 °C for 5 min. Each sample was amplified in triplicate and pooled. ExoProStar treatment (seven µL total) was performed with 0.1 µL alkaline phosphatase, 0.1 µL exonuclease, and five µL pooled PCR product, incubated at 37 °C for 30 min and 85 °C for 15 min with a Thermal Cycler (GeneAmp PCR System 2720, Thermo Fisher Scientific).

Second-round PCR (12 µL) used 1 × KAPA HiFi (KAPA Biosystems), 0.3 µM each of index primers (Table 1), and one µL of first-round PCR product. Conditions: 95 °C for 3 min, 12 cycles of 98 °C for 20 s and 72 °C for 30 s, final extension at 72 °C for 5 min. Amplicons (six µL each) were pooled and purified using SPRIselect (1:1 ratio), yielding 80 µL of eluted DNA. Concentration was measured using a Qubit 2.0 Fluorometer (Life Technologies). Further purification was performed using E-Gel (Invitrogen, USA), and fragment size (approximately 450 bp) and concentration were confirmed with an Agilent 2100 Bioanalyzer (Agilent Technologies).

### High throughput sequencing and sequence analysis

High-throughput sequencing was performed using the MiSeq system (Illumina, San Diego, CA, USA) with the MiSeq Reagent Kit v2 (300 cycles: 2 × 150 bp paired-end). This procedure was applied to all samples and as well as to all blanks, including filtration blanks, extraction blanks, and non-template blanks. Raw reads were processed as follows:

1. *Quality assessment*: Read quality was evaluated using FastQC (https://www.bioinformatics.babraham.ac.uk/projects/fastqc/) and SUGAR (Sato et al., 2014).

2. *Trimming*: Low-quality tails were trimmed using “DynamicTrim.pl” from the SOLEXAQA package (Cox, Peterson & Biggs, 2010), with a Phred score cutoff of 10 (Ewing et al., 1998).

3. *Filtering*: Forward and reverse reads were treated independently because the amplicon length was around 300 bp, which prevented merging. Custom Perl scripts were used to remove reads with abnormal lengths (expected size: 150 ± 50 bp).

4. *Primer removal*: Primer sequences were removed using TagCleaner (Schmieder et al., 2010), allowing up to five mismatches. FASTQ (Cock et al., 2010) files were converted to FASTA format.

Dereplication was performed using the ‘derep_fulllength’ command in UCLUST (Edgar, 2010), and sequences with ≥2 identical reads were retained. Chimeric sequences were identified using the ‘uchime_denovo’ command in USEARCH (Edgar, 2010). Chimeric sequences were subsequently discarded, and the remaining non-chimeric sequences were retained as amplicon sequence variants (ASVs). These ASVs were subjected to local BLASTN searches (Camacho et al., 2009) against a custom database containing *Najas* sequences from both field-collected specimens and NCBI. Species assignments were made based on ≥97% identity and an *E*-value threshold of 10^−^^5^. Reads identified as the same species from both forward (R1) and reverse (R2) reads of a sample were summed.

### Statistical analysis

We used generalized linear mixed models (GLMMs) with a zero-inflated negative binomial distribution to examine relationships between *Najas* occurrence and environmental and landscape variables.

To reduce dimensionality of landscape variables, principal component analysis (PCA) was applied to the area data of forest, paddy fields, and artificial land within 500 m of each pond. PC1 and PC2 explained 56.2% and 41.4% of the total variation, respectively. PC1 was positively correlated with paddy and artificial land and negatively with forest area. PC2 was positively correlated with paddy fields and negatively with forest and artificial land.

The GLMM included the following:

- *Response variable*: Presence/absence of *Najas* species (binomial distribution)

- *Explanatory variables*: pH, DO, EC, pond area, lengths of soil, forest, concrete shorelines, PC1, and PC2

- *Random effect*: Region (Hyogo, Awaji, Kagawa, Osaka), reflecting the four geographically distinct study areas

Species presence was determined based on both eDNA detection and direct visual observation. We first constructed a full model including all explanatory variables, followed by all possible reduced models. Model selection was based on Akaike Information Criterion corrected for small sample size (AICc). Models with ΔAICc (the difference between the AICc of the best model and each of the other models) <2 were considered plausible. Variables included in the best model were interpreted as having significant effects on *Najas* occurrence.

All analyses were conducted using R v4.4.3 and the MuMIn package v1.48.11 (R Core Team, 2025). The R script is provided as a supporting text file (Text S1). Analyses were performed on two datasets: one including all ponds and another excluding ponds with potential false positives (eDNA detected in the corresponding blank controls).

## Results

### Validation of designed primers

As a result of the *in silico* assessment of the primers we designed, Primer-BLAST indicated a strong match with the genus *Najas*, suggesting that all species within the genus could be amplified (Fig. S1). To evaluate the effectiveness of the amplified region as a DNA barcode, we calculated sequence dissimilarity among species for the target region, which ranged from 0% to 43% within *Najas*. Three species pairs—*N. marina* and *N. tenuicaulis*, *N. minor* and *N. oguraensis*, and *N. gracillima* and *N. yezoensis*—shared identical sequences and thus could not be distinguished in subsequent analyses (Table 2). *In vitro* validation confirmed successful PCR amplification for all eight tested species, demonstrating the effectiveness of the primers.

**Table 2:** Sequence dissimilarity among *Najas* species targeted by the metabarcoding marker developed in this study.

	*N. marina*	*N. tenuicaulis*	*N. minor*	*N. oguraensis*	*N. gracillima*	*N. yezoensis*	*N. graminea*	*N. orientalis*
*Najas marina*	—							
*Najas tenuicaulis*	0.000^*^	—						
*Najas minor*	0.426	0.426	—					
*Najas oguraensis*	0.426	0.426	0.000^*^	—				
*Najas gracillima*	0.400	0.400	0.309	0.309	—			
*Najas yezoensis*	0.400	0.400	0.309	0.309	0.000^*^	—		
*Najas graminea*	0.419	0.419	0.332	0.332	0.106	0.106	—	
*Najas orientalis*	0.306	0.306	0.321	0.321	0.257	0.257	0.287	—

**Notes.**

* *N. marina* and *N. tenuicaulis*, *N. minor* and *N. oguraensis*, and *N. gracollima* and *N. yeziensis* share identical sequences and cannot be distinguished.

### eDNA detection

A total of 8,990,714 raw reads were obtained from the MiSeq sequencing, including 613,002 reads from negative controls (Table S3). The raw read data have been deposited with links to BioProject accession number PRJDB37597. After quality trimming, 6,040,856 reads remained, of which 404,649 were from negative controls. Among the trimmed reads, 7,698 were identified as belonging to the target *Najas* species. Sequences that did not match the target *Najas* species were not subjected to further analysis.

Environmental DNA from *Najas* species was detected in 14 out of the 158 ponds surveyed (Fig. 1, Table S2). Specifically, detections occurred in 10 of 102 ponds in Hyogo, two of 25 in Kagawa, and two of 31 in Osaka Prefecture. Of the 14 positive ponds, 12 contained only one *Najas* sequence: *N. marina*/*N. tenuicaulis* in one pond in mainland Hyogo, *N. minor*/*N. oguraensis* in 10 ponds, and *N. gracillima*/*N. yezoensis* in one pond on Awaji Island. Two ponds contained two *Najas* sequences: *N. minor*/*N. oguraensis*, and *N. graminea* in H056, and *N. marina*/*N. tenuicaulis* and *N. minor*/*N. oguraensis* in A005. In this study, we included a total of 28 blank controls: 10 field blanks, 10 extraction blanks, and eight PCR blanks (non-template controls). Among these, two field blanks (corresponding to H001–H012 and A001–A013) and one PCR blank (O001–O025) yielded sequences assigned to *Najas* (Table S2). Notably, three of the 14 positive ponds corresponded to these blanks, indicating possible contamination. The number of reads detected for *Najas* from blanks ranged from 418 to 2,621. The sample codes are shown in the Table S2.

In addition to eDNA detection, direct visual observations of *Najas* species were recorded at five sites. At three of these sites, both eDNA and visual confirmation were obtained (Table S2).

### Results of statistical analysis

**Figure 2:**
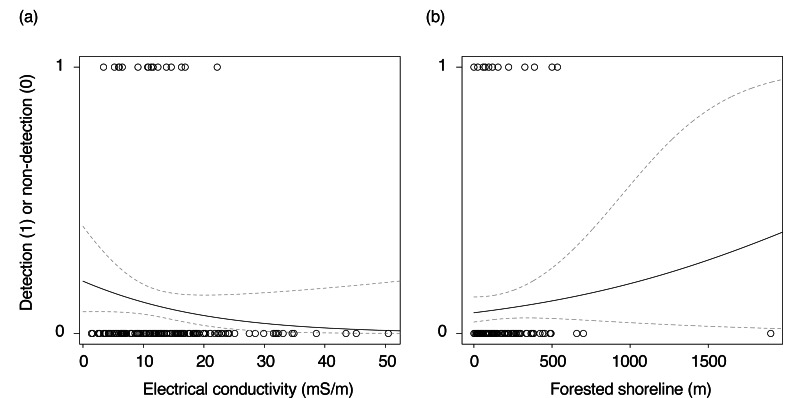
(A–B) Relationships between electrical conductivity, forested shoreline, and the detection/non-detection of *Najas*. Dashed lines indicate the range of the 95% confidence interval.

The best-fitting GLMM, as determined by the lowest AICc value, included EC and the length of forested shoreline as explanatory variables (*P* < 0.05; Table 3, Fig. 2). In this model, EC was negatively associated with *Najas* occurrence, while forested shoreline length showed a positive association.

**Table 3:** Results of GLMM and model selection Only models with delta AICc < 2 are shown.

Model	pH	DO	EC	Area	Concrete	Forest	Soil	PC1	PC2	AICc	Delta AICc
1	_	_	−0.05067	_	_	0.001469	_	_	_	109.9	0.00
2	_	−0.1008	−0.05259	_	_	0.001607	_	_	_	110.7	0.81
3	_	−0.0843	_	_	_	0.001646	_	_	_	110.8	0.90
4	_	_	−0.06978	_	_	0.001728	_	0.2376	_	110.9	1.01
5	_	_	_	_	_	0.001575	0.0006615	_	_	111.4	1.55
6	_	−0.104	−0.07394	_	_	0.001877	_	0.2538	_	111.5	1.63
7	_	_	−0.05301	_	_	0.001511	0.000714	_	_	111.5	1.65
8	_	_	_	_	_	0.001648	_	0.09222	_	111.7	1.84
9	_	_	_	_		0.001512	_	_	−0.06124	111.9	1.97

**Notes.**

DO dissolved oxygen EC electric conductivity PC1 and PC2 the first and second axes of principal component analysis

Among the nine models with ΔAICc < 2, all included forested shoreline length as a positive factor, and five included EC as a negative factor. These results suggest that both variables are important predictors of *Najas* presence.

When the analysis was repeated excluding ponds with potential false positives (*i.e.,* eDNA detected in corresponding blanks), the results were consistent: the probability of *Najas* occurrence increased with greater forested shoreline length (Table S4).

## Discussion

Our findings suggest that landscape features play a significant role in determining the distribution of rare submerged plants, as *Najas* species were detected in ponds surrounded by forested areas. Notably, eDNA of *N. minor*/*N. oguraensis* and *N. graminea* was detected at a site where one of the authors (KU) had previously observed *N. graminea*, further supporting the reliability of the eDNA-based results.

The negative correlation between *Najas* occurrence and EC indicates that these species are less likely to occur in environments with high EC. Previous studies have shown that eutrophication, driven by increased concentrations of nitrogen and phosphorus, leads to phytoplankton blooms (Phillips, Willby & Moss, 2016; Wurtsbaugh, Paerl & Dodds, 2019). This, in turn, reduces light availability in aquatic environments, limiting suitable habitats for submerged plants (Phillips, Willby & Moss, 2016; Son, Cho & Lee, 2018).

Our results also demonstrate that eDNA analysis significantly enhances the detectability of rare and endangered submerged aquatic plants, providing valuable data for conservation planning. For example, eDNA detection of *N. minor*/*N. oguraensis* in Osaka Prefecture offers new insights, as *N. minor* is listed as Data Deficient and *N. oguraensis* is listed as Critically Endangered in the local Red List. Additionally, *N. gracillima*/*N. yezoensis* eDNA was detected from Awaji Island. Although *N. gracillima* and *N. yezoensis* share identical sequences, it is likely that the detected eDNA originated from *N. gracillima*, given that *N. yezoensis* is restricted to the eastern area of Japan. Thus, this finding is quite significant because *N. gracillima* is listed as Near Threatened on the Red List 2020 in Japan. This supports the utility of eDNA for identifying populations relevant for conservation.

The GLMM results indicate that *Najas* species are more likely to occur in ponds with longer forested shorelines. This suggests that forest cover may help reduce solar radiation and maintain more stable water conditions, creating favorable environments for submerged plants. For instance, higher water temperatures can reduce dissolved oxygen and carbon dioxide levels, both of which are critical for photosynthesis. Thus, prioritizing the conservation of forest-surrounded ponds may be an effective strategy for protecting *Najas* species.

Although some blanks showed positive eDNA signals, suggesting possible contamination, the GLMM results remained consistent when these sites were excluded, indicating robustness of the findings. The detection of *Najas* sequences in several blanks highlights the challenge of contamination in eDNA studies, particularly in earlier work when laboratory protocols were less standardized. Although these findings indicate that low-level contamination cannot be completely ruled out, we have confidence in our true detections for two reasons. First, most positive samples were associated with blanks in which *Najas* DNA was not detected, reducing the likelihood that these detections were due to contamination. Second, several eDNA detections coincided with visual observations of *Najas* in the same ponds, providing independent confirmation. Nevertheless, we acknowledge that contamination risk was greater at the time this study was conducted compared to current best practices, and this limitation should be considered when interpreting our results.

In addition, we acknowledge that *Najas* species were visually observed at five sites, but eDNA was only detected at three of these locations. This discrepancy highlights a limitation of our current eDNA assay. Several factors may explain the non-detection at visually confirmed sites, including low biomass of the plants, the degradation of DNA due to environmental conditions, or sampling locations that were not close enough to the plants. It is also possible that the assay’s sensitivity may have been insufficient to detect low concentrations of eDNA. These findings underscore the importance of combining eDNA analysis with traditional field surveys, especially when monitoring rare or spatially restricted aquatic plant species. Future improvements in primer design and sampling strategies may help reduce such discrepancies and enhance the reliability of eDNA-based monitoring.

Our eDNA assay could not distinguish between several closely related species due to identical ITS sequences. For example, *N. marina* and *N. tenuicaulis* share identical sequences. *N. tenuicaulis* was described as a new species in 1935 (Miki, 1935), but subsequent taxonomic studies have been limited. Similarly, *N. minor* and *N. oguraensis* are difficult to differentiate morphologically (Kadono, 1994), and our assay could not separate them either. The same applies to *N. gracillima* and *N. yezoensis*. Given that *N. yezoensis* is restricted to eastern Japan, ecological context may help infer species identity when interpreting eDNA results.

A major challenge in primer development for *Najas* is the limited availability of nucleotide sequences in public databases. As more genetic data become available, it may be possible to identify highly variable regions that improve taxonomic resolution among closely related species.

## Conclusions

In this study, we proposed a conservation strategy for rare submerged plants by integrating habitat information from eDNA analysis, field observations, and landscape data. This approach highlights the importance of considering both habitat and surrounding environmental conditions in biodiversity conservation. eDNA-based surveys offer a safer and more efficient alternative to traditional methods, enabling large-scale monitoring of rare species such as those in the genus *Najas*. Our novel eDNA assay, capable of detecting multiple *Najas* species simultaneously, reduces survey costs and effort and may reveal previously unknown habitats. Although currently limited to *Najas*, the assay has potential for application to other rare aquatic plants. Our findings contribute new distribution data and emphasize the need to understand habitat and landscape characteristics to guide conservation priorities. Expanding eDNA methods and increasing genetic data availability will further enhance taxonomic resolution and support future conservation efforts.

## Supplemental Information

10.7717/peerj.21096/supp-1 Supplemental Information 1 Characteristics of each Najas species

10.7717/peerj.21096/supp-2 Supplemental Information 2 Water quality, landscape factors and eDNA detection / observation results of target species in each ponds

10.7717/peerj.21096/supp-3 Supplemental Information 3 The numbers of reads during each process of sequence analysis after NGS

10.7717/peerj.21096/supp-4 Supplemental Information 4 Results of data analyses by GLMM and model selection without the data of possible false positives. Only models with delta AICc ¡ 2 are shown

10.7717/peerj.21096/supp-5 Supplemental Information 5 Sequence logo of the genomic region corresponding to the primers for species of the genus *Najas* Note that the reverse side represents the reverse complementary sequence of the primer.

10.7717/peerj.21096/supp-6 Supplemental Information 6 R code for GLMM analysis used in this study

## Additional Information and Declarations

### Field Study Permissions

The following information was supplied relating to field study approvals (i.e., approving body and any reference numbers): Verbal permission for the field surveys was obtained on-site from the owner or rights holder of each pond. These informal approvals, consistent with local practices, were granted at the time of the visits. As the surveys involved non-invasive sampling in privately managed agricultural ponds, no formal written permissions or institutional approvals were required.

### Data Availability

The following information was supplied regarding data availability: The raw data and code are available in the Supplemental Files. The sequences are available at DDBJ: PRJDB37597.
