# Supplementary material for: Landscape factors influencing the distribution of rare submerged plant species: an environmental DNA (eDNA) study
Source: PeerJ. 2026 Apr 21;14:e21096. doi: 10.7717/peerj.21096 (PMC13108458; doi:10.7717/peerj.21096)
Supplement: Supplemental Information 5 — Note that the reverse side represents the reverse complementary sequence of the primer. [file peerj-14-21096-s005.pdf]

Forward

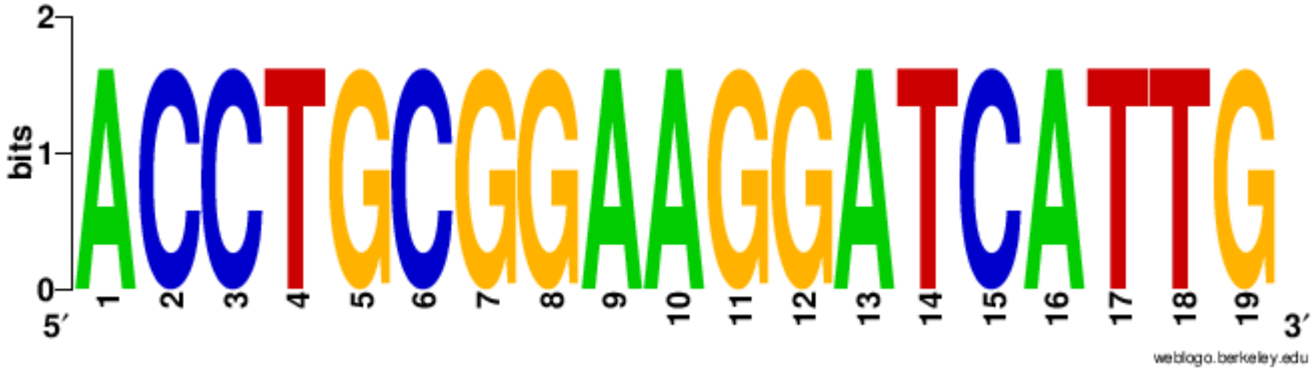


Reverse

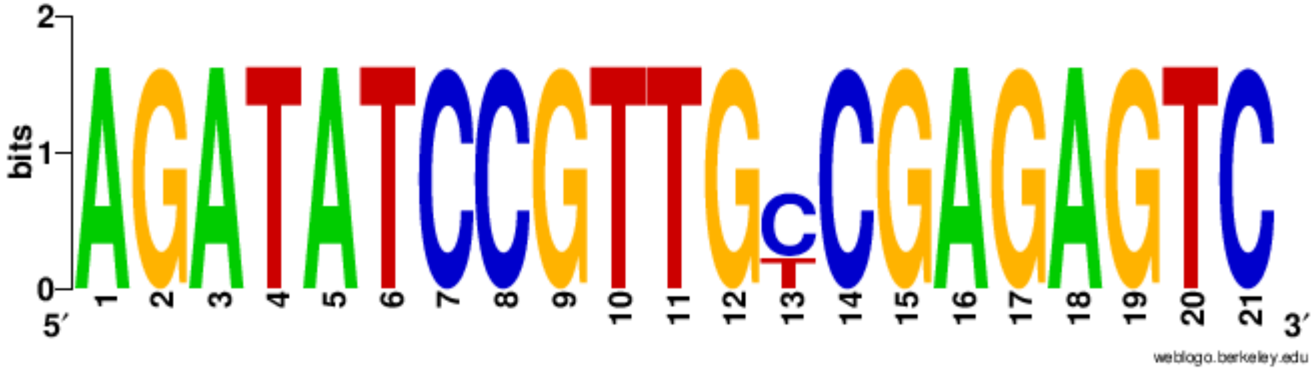


Figure S1: Sequence logo of the genomic region corresponding to the primers for species of the genus *Najas*. Note that the reverse side represents the reverse complementary sequence of the primer.
